# A Coordination Network Featuring Two Distinct Copper(II) Coordination Environments for Highly Selective Acetylene Adsorption

**DOI:** 10.1002/chem.202201188

**Published:** 2022-07-28

**Authors:** Magdalene W. S. Chong, Stephen P. Argent, Florian Moreau, William J. F. Trenholme, Christopher G. Morris, William Lewis, Timothy L. Easun, Martin Schröder

**Affiliations:** ^1^ School of Chemistry University of Nottingham University Park Nottingham NG7 2RD UK; ^2^ School of Chemistry The University of Manchester Oxford Road Manchester M13 9PL UK; ^3^ School of Chemistry Cardiff University Main Building, Park Place Cardiff CF10 3AT UK

**Keywords:** adsorption, copper, crystal engineering, metal-organic frameworks, microporous materials, supramolecular chemistry

## Abstract

Single crystals of 2D coordination network {Cu_2_ **L**_2_ ⋅ (DMF)_3_(H_2_O)_3_}_*n*_ (**1‐DMF**) were prepared by reaction of commercial reagents 3‐formyl‐4‐hydroxybenzoic acid (H_2_ **L**) and Cu(NO_3_)_2_ in dimethylformamide (DMF). The single‐crystal structure shows two distinct Cu(II) coordination environments arising from the separate coordination of Cu(II) cations to the carboxylate and salicylaldehydato moieties on the linker, with 1D channels running through the structure. Flexibility is exhibited on solvent exchange with ethanol and tetrahydrofuran, while porosity and the unique overall connectivity of the structure are retained. The activated material exhibits type I gas sorption behaviour and a BET surface area of 950 m^2^ g^−1^ (N_2_, 77 K). Notably, the framework adsorbs negligible quantities of CH_4_ compared with CO_2_ and the C_2_H_*n*_ hydrocarbons. It exhibits exceptional selectivity for C_2_H_2_/CH_4_ and C_2_H_2_/C_2_H_*n*_, which has applicability in separation technologies for the isolation of C_2_H_2_.

## Graphical abstract

**A 2D coordination network** was synthesised from commercial reagents and displays two distinct coordination environments of copper to carboxylate and salicylaldehydato moieties of the ligand. Solvent exchange of the synthesized network shows retention of framework connectivity coupled with unanticipated framework flexibility to accommodate tetrahydrofuran. The activated framework selectively adsorbs CO_2_ and C_2_H_*n*_ hydrocarbons over CH_4_.

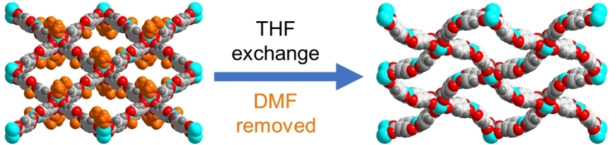

## Introduction

Porous materials have garnered much industrial interest for the exploitation of their gas sorption properties.^[1]^ Gas storage is an obvious application for materials exhibiting permanent porosity, which is reflected in the breadth of research into the optimisation of gas adsorption capacities of metal‐organic frameworks (MOFs).^[2]^ MOFs are highly crystalline coordination materials that comprise metal‐based centres (consisting of ions or clusters) connected by multidentate organic ligands. The ability to design, tune the properties of, and fully structurally characterise MOFs facilitates their applicability for selective gas adsorption and separation.^[3]^

Strategic design of MOFs to target selective adsorption of small molecules such as CO_2_, CH_4_ and the C_2_H_*n*_ hydrocarbons commonly focusses on exploiting differences in the physical and chemical properties of these adsorbates (Table 1), which currently pose high financial and energy costs to separate industrially.^[4]^ Similarities in the physical‐chemical properties of CO_2_ and C_2_H_2_ result in many MOFs adsorbing these two substrates preferentially over many hydrocarbons.^[6a]^ The smaller kinetic diameters of CO_2_ and C_2_H_2_ allow MOFs with narrow pore apertures to selectively adsorb these smaller substrates.^[7]^ Additionally, the higher quadrupole moments of CO_2_ and C_2_H_2_ (Table 1) can facilitate stronger interactions with the pore surfaces of a MOF,^[8]^ particularly when polar functional groups are incorporated to enhance adsorption.^[6]^ Another distinction of quadrupolar adsorbates is that they are also able to favourably bind to open metal sites within the MOFs via preferential interactions of their π‐electrons with the metal sites, resulting in selectivity for CO_2_ ^[9]^ and C_2_H_2_.[^8c^, ^10^] This has been highlighted in particular with MOFs featuring open Cu(II) coordination sites at the metal nodes^[11]^ and those incorporating metal sites via salen‐based ligands,^[12]^ with the most relevant example being the use of a Cu(II) salen pillaring ligand alongside Zn(II) nodes in mixed metal MOF M'MOF‐20a that incorporates open Cu(II) sites available for guest binding, reported by Chen et al. in 2012.^[12a]^

**Table 1:** Physical‐chemical properties of selected natural gas components.^[a]^

	b.p. [K, 1 atm]^[b]^	*μ*×10^18^ [esu cm]^[c]^	*Θ*×10^26^ [esu cm^2^]^[d]^	*σ* [Å]^[e]^
N_2_	77.35	0	1.52	3.6–3.8
CO_2_	216.55	0	4.30	3.3
CH_4_	111.66	0	0	3.8
C_2_H_6_	184.55	0	0.65	4.4
C_2_H_4_	169.42	0	1.50	4.2
C_2_H_2_	188.40	0	4.71	3.3

[a] Data from References [3d] and [5]. [b] Boiling point. [c] Dipole moment. [d] Quadrupole moment. [e] Kinetic diameter.

In this paper we describe a similar method whereby commercially available 3‐formyl‐4‐hydroxybenzoic acid (H_2_ **L**) serves as a bidentate ligand upon deprotonation of the salicylaldehyde and carboxylic acid moieties. The salicylaldehydato motifs from two units of **L** ^2−^ are able to complex Cu(II) cations^[13]^ whilst concomitant coordination by the carboxylate moiety^[14]^ leads to a continuous 2D network. This approach affords a high density of open Cu(II) sites as the two coordinating groups on the linker are separated by only a single phenyl ring. Furthermore, the use of readily available reagents is advantageous for future scalability of the product.

## Results and Discussion

**Figure 1:**
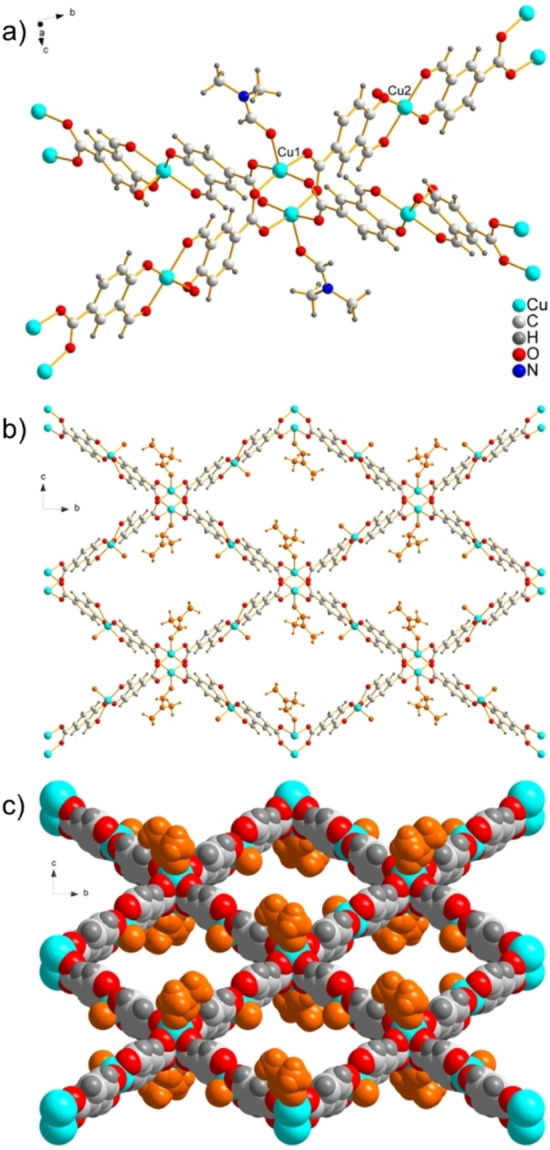
a) View of a fragment of the single crystal X‐ray structure of **1‐DMF** showing connectivity of a Cu(II) paddlewheel, coordinated by four carboxylate moieties of **L** ^2−^, to four other paddlewheels. Two units of **L** ^2−^ coordinated at the salicylaldehydato moiety to another Cu(II) cation are situated between each paddlewheel. DMF occupies the apical position of Cu1 at the Cu(II) paddlewheels. Only the oxygen component of the disordered DMF bound to Cu2 has been modelled. Channels in **1‐DMF** viewed down the crystallographic *a*‐axis: b) ball and stick model, and c) space filling model with DMF solvent molecules in orange.

An equimolar mixture of H_2_ **L** and copper nitrate were dissolved in *N*,*N*‐dimethylformamide (DMF) in the presence of hydrochloric acid. Solvothermal heating of the green solution at 85 °C in a sealed vessel yielded green block single crystals of **1‐DMF**, which were shown by single crystal X‐ray diffraction and elemental analysis to have the formula {Cu_2_ **L**_2_ ⋅ (DMF)_3_(H_2_O)_3_}_*n*_. **1‐DMF** crystallises in space group *I*2/*m* (Table 2) and comprises layers of 2D networks with **sql** topology,^[15]^ in which **L** ^2−^ is bound to the Cu(II) cations in two distinct coordination environments (Figure 1).

**Table 2:** Summary of selected single crystal data for three forms of **1**; as synthesised **1‐DMF**, and solvent exchanged with ethanol and THF **1‐EtOH** and **1‐THF** respectively. The final column is the indexing parameters from the PXRD of **1‐DMF**.

	**1‐DMF**	**1‐EtOH**	**1‐THF**	PXRD (**1‐DMF**)
Crystal system	monoclinic	monoclinic	monoclinic	monoclinic
Space group	*I*2/*m*	*I*2/*m*	*P*2_1_/*n*	*I*2/*m*
*a* [Å]	7.7935(5)	6.8089(9)	8.0708(9)	7.2888(6)
*b* [Å]	24.540(2)	25.808(3)	25.437(3)	24.604(3)
*c* [Å]	16.1842(16)	18.208(4)	14.115(3)	15.8857(8)
*β* [°]	92.097(8)	89.805(16)	96.986(13)	100.751(7)
Volume [Å^3^]	3093.2(4)	3199.5(8)	2876.4(7)	2798.8(5)
*Z*	4	4	4

Carboxylate groups from four ligands **L** ^2−^ form {Cu_2_(RCO_2_)_4_(DMF)_2_} paddlewheel units with symmetry related pairs of Cu(II) cations (Cu1 in Figure 1a), a common motif found in Cu(II)‐carboxylate MOFs.^[14]^ Salicylaldehydato moieties from two ligands **L** ^2−^ coordinate to Cu(II) cation Cu2 in a bidentate fashion, resulting in a salen‐like motif^[13]^ in which the square pyramidal coordination geometry is capped by a disordered solvent molecule. The four‐connected Cu(II) paddlewheel units thus act as nodes and the two‐connected square pyramidal bis(salicylaldehydato)‐Cu(II) motifs as struts in the (4,4)‐connected net of the aforementioned **sql** topology. The (4,4)‐connected nets are rhombus shaped, having edges 19.0 Å long (measured between centroids of consecutive paddlewheels) and internal angles of 88 ° and 99 °. The 2D networks are stacked with the paddlewheel nodes 7.8 Å apart and with an offset that allows the coordinated DMF solvent molecules to protrude into the windows of the networks above and below. The stacking results in continuous channels running through the (4,4)‐connected net apertures in the direction of the crystallographic *a*‐axis. In addition to the DMF molecules bound to the paddlewheels, disordered bis(salicylaldehydato)‐Cu(II) bound solvent molecules also protrude into the channels. Only the oxygen atom of the bis(salicylaldehydato)‐Cu(II) bound solvent entity could be crystallographically modelled, however, residual electron density treated by PLATON SQUEEZE^[16]^ indicated the species are a mixture of DMF and water disordered by symmetry over either side of the bis(salicylaldehydato)‐Cu(II) plane. The channels have a width of 11.7 Å (taking into account the van der Waals radius of Cu(II)) measured between bis(salicylaldehydato)‐Cu(II) cations from adjacent nets (a shorter distance than that between Cu(II) cations of the same net owing to the angle between the plane of the nets and the direction of the channels).

A batch of as‐synthesised **1‐DMF** crystals was solvent exchanged by immersion in ethanol for seven days; subsequent single crystal X‐ray analysis of the resulting material **1‐EtOH** confirmed retention of crystallinity and a new formula {Cu_2_ **L**_2_ ⋅ (EtOH)_3.5_}_*n*_. The new phase remains in space group *I*2/*m* with an increased unit cell volume caused by a flexing of the rhombus net (Table 2). The structure of **1‐EtOH** retains **sql** topology, however, as a result of weaker diffraction than for **1‐DMF** only a partial model of the paddlewheel bound ethanol molecule could be developed and no solvent could be modelled at the bis(salicylaldehydato)‐Cu(II) axial site Cu2. The presence of disordered solvent at the axial site of Cu2 cannot be ruled out and given the large void adjacent to the site is indeed likely. Despite the diffraction deficiencies of **1‐EtOH**, the retention of crystallinity and change in unit cell parameters clearly demonstrate the preservation of the material connectivity after solvent exchange. Rhombus net flexing is commonly seen as a component of the “breathing” behaviour of some 3D “wine‐rack structures”, such as MIL‐53;^[17]^ observing overall retention of crystallinity in this 2D network is perhaps less anticipated.

**Figure 2:**
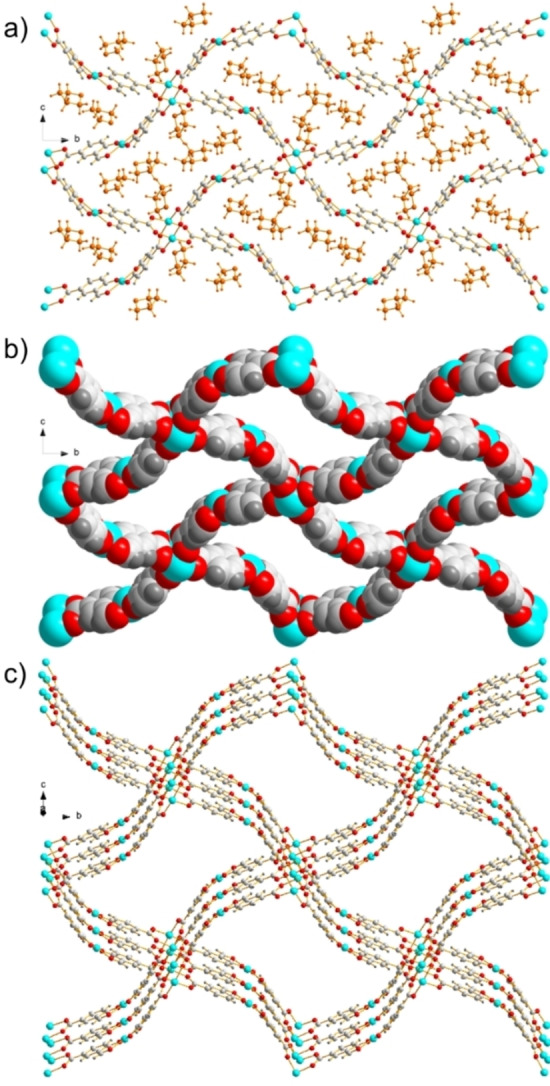
Channels in **1‐THF** viewed down the crystallographic *a*‐axis: a) ball and stick model with THF molecules in orange, b) space filling model with THF molecules omitted to show potential porosity upon desolvation, and c) slightly offset from the crystallographic *a*‐axis to show stacking of layers.

Solvent exchange of as‐synthesised **1‐DMF** single crystals was also carried out by immersion in tetrahydrofuran (THF) for seven days to give **1‐THF** (Figure 2). X‐ray analysis of the resulting phase revealed complete exchange of all solvent sites with THF molecules, giving a new formula {Cu_2_ **L**_2_ ⋅ (THF)_3_}_*n*_. **1‐THF** retains the same network connectivity and overall Cu(II) coordination environments as **1‐DMF**, however, the structure is now solved in space group *P*2_1_/*n*. The network demonstrates further flexibility to accommodate the incoming THF solvent molecules; previously straight edges of the rhombus apertures in the (4,4)‐connected nets are now bent at an angle of 4.4 ° measured between centroids of two paddlewheel nodes and the interstitial linking Cu(II) cation. Refinement of symmetry related THF molecules on the paddlewheels and on both sides of the bis(salicylaldehydato)‐Cu(II) indicates all sites are fully occupied (Figure 2a). The pairs of THF molecules on each side of the bis(salicylaldehydato)‐Cu(II) are coordinated via long Cu2⋅⋅⋅O contacts of 2.48(1) Å and 2.50(1) Å, in contrast to the shorter distance observed for single DMF molecules on only one side of the Cu(II) cation in **1‐DMF**. These three single crystal X‐ray structures give crystallographic evidence of the retention of crystallinity of single crystals of **1‐DMF** during solvent exchange and that, despite its flexibility, the coordination motifs of **L** ^2−^ bound to Cu(II) cations are maintained during exposure to different solvents.

A powder X‐ray diffraction (PXRD) pattern of a bulk sample of **1‐DMF** was measured, the data were indexed and a Pawley refinement^[18]^ carried out (Figures S1 and S2) giving a monoclinic unit cell in space group *I*2/*m* (Table 2), consistent with the single crystal X‐ray structure. Notably, the unit cell volume of **1‐DMF** measured at room temperature by PXRD is smaller than that observed in the 120 K single crystal structure; this may either be a result of temperature‐dependent flexibility or due to partial desolvation during the PXRD experiment. The PXRD pattern of **1‐EtOH** shows some loss of crystallinity. We ascribe this to the higher volatility of ethanol compared with DMF, therefore the interactions holding the sheets of **1** may be disrupted, lowering the crystallinity of the sample. Additionally, the apparent flexibility of **1** is likely to impact the apparent quality of the PXRD, as observed for other flexible MOFs.^[19]^ We also recorded the PXRD pattern of **1**, the desolvated form of **1‐EtOH**, before and after completing a series of gas sorption experiments (see below) and confirmed that after the initial loss of crystallinity on activation no significant further loss of crystallinity was observed (Figure S1).

Thermogravimetric analysis (TGA) was performed on **1‐DMF** and **1‐EtOH** (Figure S3) under a nitrogen atmosphere. Two distinct mass losses are observed in the TGA of **1‐DMF** corresponding to the loss of unbound and coordinated solvent respectively; no further mass loss is observed until 300 °C. In **1‐EtOH**, only one mass loss event related to expulsion of solvent is observed, which can again be attributed to the removal of this more volatile guest.

**Figure 3:**
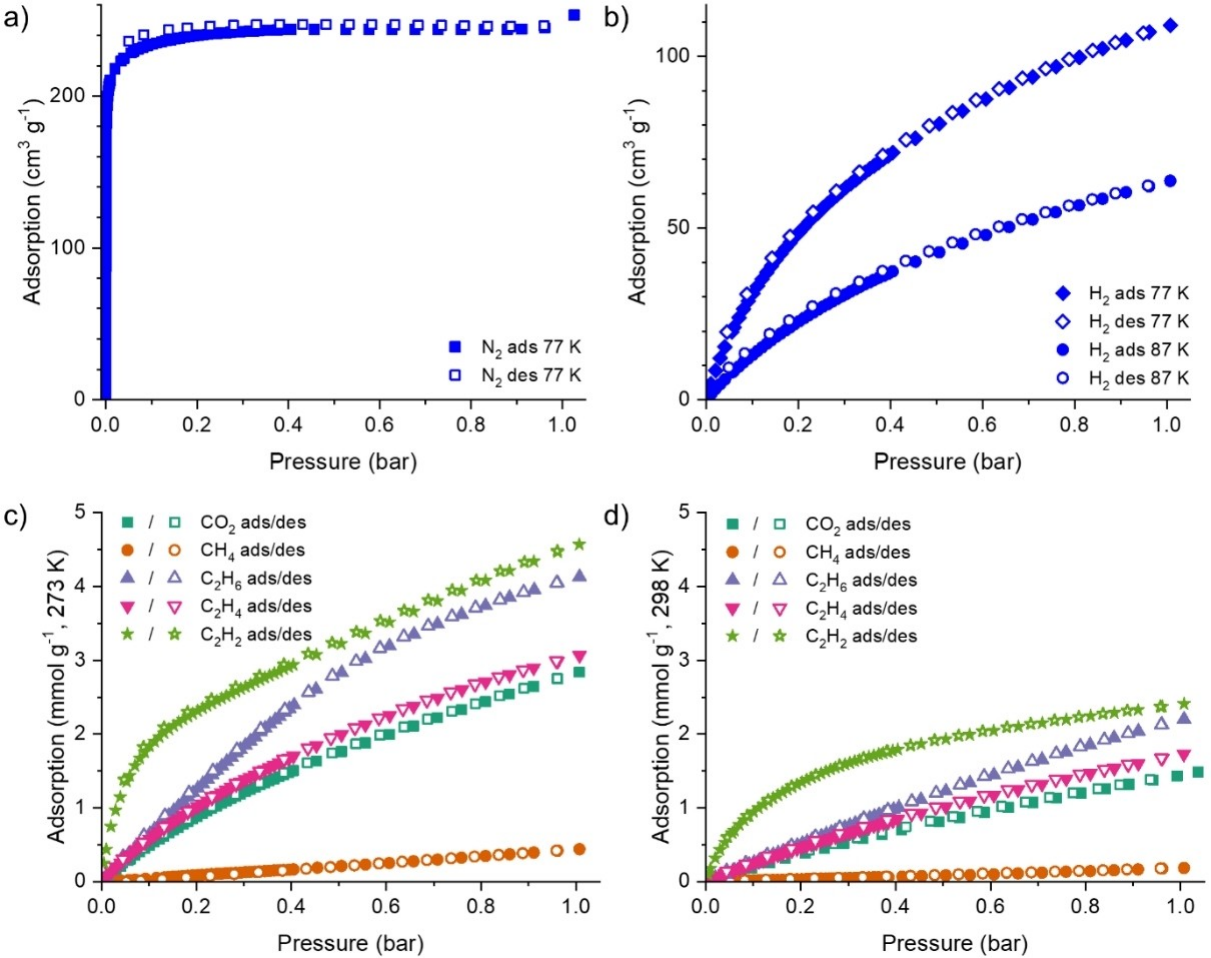
Adsorption and desorption isotherms of **1** for: a) N_2_, b) H_2_, and CO_2_, CH_4_ and the C_2_H_*n*_ hydrocarbons at c) 273 K and d) 298 K.

The permanent porosity of **1** (the desolvated form of **1‐EtOH**) was confirmed by a N_2_ isotherm (Figure 3a), which exhibits reversible type I adsorption.^[20]^ The N_2_ adsorption at 1.0 bar and 77 K is 253 cm^3^ g^−1^, with the Brunauer‐Emmett‐Teller (BET) surface area of **1** calculated to be 948±1 m^2^ g^−1^. By comparison, the solvent accessible volume from the crystallographic data of fully desolvated **1‐DMF** calculated using PLATON SQUEEZE^[16]^ is 54 %, corresponding to a pore volume of 0.548 cm^3^ g^−1^. The micropore volume from the N_2_ isotherm of **1** is 0.324 cm^3^ g^−1^; the difference between the crystallographically expected free volume of desolvated **1‐DMF** and experimental isotherm measured volume of **1** is attributed to framework flexibility upon desolvation. A non‐local density functional theory (NLDFT) pore diameter of 11 Å was modelled from the N_2_ isotherm data.

H_2_ sorption isotherms of **1** were measured at 77 K and 87 K (Figure 3b) and also exhibited reversible type I behaviour, consistent with the N_2_ isotherm. The amount of H_2_ adsorbed at 1.0 bar is 109 cm^3^ g^−1^ (77 K) and 64 cm^3^ g^−1^ (87 K). The H_2_ adsorption at 1.0 bar and 77 K corresponds to 0.98 wt %, which is comparable to other reported MOFs featuring open metal sites^[2c]^ but is not industrially competitive for H_2_ storage.

Adsorption and desorption isotherms for CO_2_, CH_4_ and the C_2_H_*n*_ hydrocarbons in **1** were measured at 273 K and 298 K (Figures 3c and 3d). The quantity adsorbed at 1.0 bar for the different adsorbates is summarised in Table 3. The isotherms all exhibit reversible type I behaviour (Figure 3), again confirming that **1** is a microporous material. The absence of desorption hysteresis suggests the diffusion of the adsorbed material out of **1** is not hindered by interactions between the adsorbate and the network or framework structural changes. The channels are much larger than the gases selected, therefore sieving effects, such as the geometric advantage for linear molecules (CO_2_ and C_2_H_2_) to penetrate into a framework, are unlikely to influence the adsorption properties.

**Table 3:** Quantity adsorbed by **1** of studied adsorbates at 1.0 bar (273 K or 298 K), *Q*_st_ calculated for **1** at zero loading for the different adsorbates and *k_H_* values obtained (273 K or 298 K) for the different adsorbates.

	H_2_	CO_2_	CH_4_	C_2_H_6_	C_2_H_4_	C_2_H_2_
Adsorption, 273 K [mmol g^−1^]	–	2.86	0.44	4.15	3.08	4.55
Adsorption, 298 K [mmol g^−1^]	–	1.47	0.18	2.18	1.74	2.41
*Q*_st_ [kJ mol^−1^]	6.0	27.1	–	25.7	27.6	21.4
*k_H_*, 273 K [×10^−7^, mol g^−1^ Pa^−1^]	–	0.56	–	0.69	0.71	5.60
*k_H_*, 298 K [×10^−7^, mol g^−1^ Pa^−1^]	–	0.21	–	0.27	0.28	2.06

Adsorption of CH_4_ by **1** (Figure 3 and Table 3) is very low, with the quantity of CH_4_ adsorbed an order of magnitude lower than the other gases investigated. This may be partially attributed to its comparatively low molecular mass, but more significantly, CH_4_ is the only non‐quadrupolar adsorbate studied herein (Table 1). The CH_4_ adsorption is 0.71 wt % (273 K) and 0.29 wt % (298 K), which is very low compared with other microporous frameworks.^[2e]^

C_2_H_6_ has a small quadrupole moment (Table 1), which may afford some additional interaction with the open Cu(II) sites in **1** compared with CH_4_. Additionally, stronger van der Waals forces of the larger C_2_H_6_, increasing the strength of interactions compared to CH_4_, may promote greater adsorption of C_2_H_6_ into the network. This is observed experimentally (Table 3). The C_2_H_6_ isotherms (Figure 3) are linear until 0.4 bar, accounting for almost half of the uptake. At 1.0 bar, the C_2_H_6_ uptake at 273 K approaches saturation.

The isotherm profiles for CO_2_ and C_2_H_4_ are similar (Figure 3). For both adsorbates the steepest adsorption region in the isotherms is between 0 and 0.2 bar (Figure S4), with total uptake in this region of ca. 3 mmol g^−1^ (273 K, 1.0 bar), but neither of these are at saturation.

The profiles of the C_2_H_2_ isotherms are very different to the other adsorbates (Figure 3). At low pressure there is a steep increase in the uptake of C_2_H_2_; 30 % of the total uptake at 1.0 bar is observed by 0.06 bar, indicating very high affinity for C_2_H_2_ at low pressures. Uptake of C_2_H_2_ at 1.0 bar is higher than the other two C_2_H_*n*_ hydrocarbons, which may be partially attributed to its comparatively smaller size. Furthermore, π‐interactions between C_2_H_2_ and the framework also likely contribute to an increase in the uptake capacity.

The isosteric heat of adsorption (*Q*_st_) was calculated for all the adsorbates apart from CH_4_ (Table 3) by comparing the adsorption at two temperatures using the virial method.^[21]^ A satisfactory fit could not be obtained for the CH_4_ adsorption data (Figure S7), which may be attributed to the low uptake of CH_4_ by **1** in this pressure range. The *Q*_st_ for CO_2_ (27.1 kJ mol^−1^) is between the expected range^[4e]^ of 20 to 50 kJ mol^−1^ and decreases with increased loading (Figure S6). A high *Q*_st_ is good for adsorption but not always desirable for separation purposes because of the large energy requirement associated with regeneration (desorption) of the material.^[2b]^ The *Q*_st_ of C_2_H_2_ is lower than the other C_2_H_*n*_ hydrocarbons and CO_2_, but increases as a function of loading (Figure S10) whilst *Q*_st_ decreases with loading for the other substrates. Therefore, in the case of C_2_H_2_, it is likely that there are also strong adsorbate‐adsorbate interactions.

The selectivity properties of **1** were predicted by application of Henry's law and the ideal adsorbed solution theory (IAST) to the single‐component adsorption isotherm data. The IAST method is considered the benchmark protocol for predicting selectivities in multicomponent mixtures from single component isotherms,^[22]^ whilst the application of Henry's law is facile in comparison and offers an indicator of the expected adsorption selectivities at low loading. However, both models are limited by the absence of measures to account for adsorbate‐adsorbate interactions.

The calculations herein for Henry's constant, *k*_H_, proceed similarly to the calculation of *Q*_st_ whereby the adsorption isotherm data for each gas are fitted to a virial equation^[23]^ and the coefficient *A*_0_, related to adsorbate‐adsorbent interactions,^[24]^ is used to calculate *k*_H_ (Table 3).^[25]^ Again, this was not possible for CH_4_ as the adsorption in this pressure range was too low to perform a suitable fitting (Figure S11), therefore a quantitative selectivity with respect to CH_4_ cannot be obtained using *k*_H_. Where a *k*_H_ value has been calculated, the selectivity equates to the ratio of the *k*_H_ values for the adsorbates concerned.

The IAST method enables modelling of mixed gas adsorption from single component isotherms and thus a prediction of selectivity.^[26]^ The experimental single‐component adsorption data is fitted to an isotherm model to obtain the required parameters,^[27]^ with the Langmuir‐Freundlich model used herein.^[28]^ Full details of the fittings and modelling can be found in the Supporting Information. The selectivity was calculated from the predicted isotherms for 50 : 50 binary systems. For instances where the major assumptions of the IAST model[^2b^, ^22^] do not hold, the accuracy is reduced and only a qualitative prediction may be extrapolated; this applies especially to C_2_H_2_/CH_4_ in this work.

Overlay of the CO_2_ and CH_4_ adsorption isotherms (Figure 3) indicates a preference for adsorption of CO_2_ by **1**, reflected by the steeper gradient of CO_2_ uptake, especially at low loading. Calculations by the IAST method indicate respectable CO_2_/CH_4_ selectivity by **1** of 24 : 1 at 273 K (Figure S15). We attribute this to a greater affinity for quadrupolar CO_2_ over non polar CH_4_. Adsorbate interactions with the internal surfaces of **1** are also likely to be enhanced for CO_2_ vs. CH_4_ due to its smaller size and linear geometry.

Similarly, overlay of the isotherms for all of the C_2_H_*n*_ hydrocarbons and CH_4_ (Figure 3) indicates very low CH_4_ uptake in comparison to the other adsorbates at both 273 K and 298 K, with differences in the uptake profiles suggesting good C_2_H_*n*_/CH_4_ selectivity. As a *k*_H_ value has not been obtained for CH_4_ uptake, quantitative selectivities of C_2_H_*n*_/CH_4_ have not been obtained via Henry's law. Analysis by the IAST method confirms C_2_H_*n*_/CH_4_ selectivity (Figure S16), with the highest selectivity values determined for C_2_H_2_/CH_4_. At 1.0 bar and 273 K a selectivity of ca. 6000 : 1 is obtained for C_2_H_2_/CH_4_; this unusually high value strongly suggests that the assumptions of the IAST model have not been upheld. This may be attributed to both a relatively low CH_4_ uptake up to 1.0 bar and adsorbate‐adsorbate interactions of C_2_H_2_, which is in agreement with the *Q*_st_ for C_2_H_2_. Additionally, a qualitative interpretation supports the assessment that **1** demonstrates highest selectivity for C_2_H_2_/CH_4_. The *k*_H_ values for C_2_H_2_ exceed C_2_H_6_ and C_2_H_4_ by an order of magnitude (Table 3), thus if *k*_H_ could be determined for CH_4_, the selectivity for C_2_H_2_/CH_4_ would be proportionally greater than C_2_H_6_/CH_4_ and C_2_H_4_/CH_4_. Notably C_2_H_2_ has the potential for specific interactions with the internal surfaces of the network (at both the Cu(II) sites and phenyl rings of **L** ^2−^) that are weaker/not present for CH_4_.

Selectivity for C_2_H_*n*_/CH_4_ may be generally attributed to stronger van der Waals interactions between larger substrates and the framework. The values calculated by the IAST method for the selectivity of C_2_H_6_/CH_4_ (65 : 1 at 273 K) and C_2_H_4_/CH_4_ (34 : 1 at 273 K) by **1** are reasonable, in contrast to the case of C_2_H_2_/CH_4_ (ca. 6000 : 1 at 273 K). At both temperatures, the selectivity of C_2_H_4_/CH_4_ is greater than C_2_H_6_/CH_4_ at low loadings (Figure S16). This may be due to the higher quadrupole moment of C_2_H_4_ enabling stronger interactions with open Cu(II) sites and its slightly smaller size and linear geometry enhancing interactions with the internal surfaces of the network. At both temperatures, there is an intersection pressure above which the selectivity of C_2_H_6_/CH_4_ exceeds that of C_2_H_4_/CH_4_. At higher pressures van der Waals interactions may dominate both adsorbate‐adsorbent and adsorbate‐adsorbate interactions, thus benefitting C_2_H_6_ in comparison to C_2_H_4_.

The adsorption of C_2_H_6_ exceeds C_2_H_4_ before 0.05 bar, but the profiles for the uptake of both adsorbates are very similar at low pressure (Figure S4). Analysis by the IAST method shows that the selectivity for C_2_H_6_/C_2_H_4_ does not exceed 1.5 at either temperature (Figure S17), thus precluding utility of **1** as a separation medium for these two adsorbates. This is consistent with values obtained by comparison of the *k*_H_ values (Table S5), which indicate a lack of selectivity between these two gases. Selectivities for C_2_H_2_/C_2_H_*n*_ (*n*=4, 6) appear relatively similar by the IAST method (Figure S18), which is consistent with selectivities of ca. 8 : 1 (273 K) derived from Henry's law in both cases (Table S5). The selectivities at very low pressure (<0.15 bar) determined by the IAST method are greater for C_2_H_2_/C_2_H_6_, but at increased pressures the selectivity is greater for C_2_H_2_/C_2_H_4_. C_2_H_2_ is clearly an excellent match for the network with favourable electronic and geometric complementarities.

The gas adsorption selectivities of **1** for C_2_H_2_/CO_2_ (10.0 : 1 at 273 K; 9.8 : 1 at 298 K) are slightly higher than those reported for the related M'MOF‐20a (6.2 : 1 at 273 K; 5.1 : 1 at 295 K) which incorporates Cu(II) salen moieties.^[12a]^ The C_2_H_2_/C_2_H_*n*_ selectivity of M'MOF‐20a is not reported, but network **1** has excellent selectivity for C_2_H_2_/C_2_H_*n*_ which we attribute in part to the high concentration of available Cu(II) binding sites within the network.

## Conclusion

Solvothermal reaction of H_2_ **L** with copper nitrate yielded green single crystals of **1‐DMF**, which comprises layers of 2D networks. Contrasting binding of **L** ^2−^ to Cu(II) at both the salicylaldehydato and carboxylate moieties results in two distinct coordination environments, both of which have the capacity to undergo solvent exchange and removal. The network structure is retained when the single crystals are solvent exchanged with ethanol and THF, with framework flexibility demonstrated by changes in the unit cell dimensions of the crystal structure. In **1‐THF** full occupancy THF molecules are located at all available Cu(II) sites and the network distorts and contracts in volume to accommodate the solvent molecules. Channels run through the crystallographic *a*‐axis of the structure in all cases and the porosity of **1** was confirmed by gas sorption experiments.

Activated material **1** maintains porosity, exhibiting type I adsorption and having a BET surface area of 948±1 m^2^ g^−1^ (N_2_). Comparison of measured isotherms indicate **1** adsorbs very low amounts of CH_4_ compared to CO_2_ and the C_2_H_*n*_ hydrocarbons up to 1.0 bar, which is attributed to the inability of non‐quadrupolar CH_4_ to interact strongly with the Cu(II) sites of **1**. The low affinity for CH_4_ and exceptional selectivity for C_2_H_2_ has applications in purification of these gases. The preparation of **1** from commercially available precursors is simple and reproducible, thus it is an ideal candidate for scalability in future investigations.

## Experimental Section

All chemicals were obtained from commercial sources and used as received without further purification. Elemental microanalysis was performed using an Exeter Analytical CE 440 elemental analyser. Single crystals were extracted directly from the mother liquor, mounted under a film of Fomblin perfluoropolyether on a MiteGen Micromount and flash frozen under a cold stream of N_2_. Diffraction data were collected on an Agilent Technologies SuperNova diffractometer with a microfocus Cu X‐ray source, using CrysAlis PRO^[29]^ for collecting frames of data. The raw data were reduced and corrected for Lorentz and polarisation effects using CrysAlis PRO;^[29]^ corrections for the effects of adsorption were applied using a numerical absorption correction based on Gaussian integration over a multifaceted crystal model. The structures were solved by direct methods using ShelXS^[30]^ or ShelXT^[31]^ and refined with the ShelXL^[32]^ refinement package using full matrix least squares minimisation. Details of the crystal structure refinements can be found in the Supporting Information. PXRD data were collected on a PANalytical X'Pert PRO diffractometer with a Cu source (λ=1.5432 Å) and reflection‐transmission spinner PW3064; a Pawley refinement^[18]^ of the data was performed using TOPAS^[33]^ to extract the unit cell parameters. TGA data was measured using a Perkin Elmer Pyris 1 TGA thermogravimetric analyser. Samples for gas adsorption measurements were outgassed on a Micromeritics Smart VacPrep at 100 °C and 1.0 mm Hg s^−1^ for 15 h prior to analysis with a Micromeritics 3Flex surface characterisation analyser using research grade gas as received. Temperatures of 77 K and 87 K were obtained using liquid nitrogen and liquid argon baths respectively. A Julabo ED heating immersion circulator was employed for temperature control to perform measurements at 273 K and 298 K.

**Synthesis of 1‐DMF**: To a solution of Cu(NO_3_)_2_ ⋅ 3H_2_O (29 mg, 0.12 mmol) and H_2_ **L** (20 mg, 0.12 mmol) dissolved in DMF (2 ml), HCl (2 M, 2 drops) was added. The green solution was sealed in a pressure tube and heated at 85 °C for 48 h, affording green crystals which were washed with DMF. This synthesis was repeated in 5 pressure tubes to obtain sufficient sample for gas sorption experiments (total mass 109 mg, 0.15 mmol, 25 %). Elemental analysis calcd (%) for C_25_H_35_Cu_2_N_3_O_14_: C 41.3, H 4.8, N 5.8; found C 41.7, H 4.3, N 5.4.

**Preparation of 1‐EtOH and 1‐THF**: Single crystals of **1‐DMF** were immersed in the exchange solvent (ethanol or THF) and the solvent refreshed daily over seven days.

Deposition Number(s) 1558207 (**1‐DMF**), 1558208 (**1‐EtOH**), and 1558209 (**1‐THF**) contain(s) the supplementary crystallographic data for this paper. These data are provided free of charge by the joint Cambridge Crystallographic Data Centre and Fachinformationszentrum Karlsruhe Access Structures service.

## Conflict of interest

The authors declare no conflict of interest.

1

## Supporting information

As a service to our authors and readers, this journal provides supporting information supplied by the authors. Such materials are peer reviewed and may be re‐organized for online delivery, but are not copy‐edited or typeset. Technical support issues arising from supporting information (other than missing files) should be addressed to the authors.

Supporting Information

## Data Availability

The data that support the findings of this study are available in the supplementary material of this article.
