# Supplementary material for: A Coordination Network Featuring Two Distinct Copper(II) Coordination Environments for Highly Selective Acetylene Adsorption
Source: Chemistry. 2022 Jul 28;28(52):e202201188. doi: 10.1002/chem.202201188 (PMC9545019; doi:10.1002/chem.202201188)
Supplement: Supplementary file 1 — Supporting Information [file CHEM-28-0-s001.pdf]

Chemistry–A European Journal

Supporting Information

A Coordination Network Featuring Two Distinct Copper(II) Coordination Environments for Highly Selective Acetylene Adsorption

Magdalene W. S. Chong, Stephen P. Argent, Florian Moreau, William J. F. Trenholme, Christopher G. Morris, William Lewis, Timothy L. Easun,* and Martin Schröder*

Table of Contents

1) Crystallographic data **2**
Table S1

2) Analysis of bulk material **3**
Figures S1 – S4

3) Q_{st} calculations **5**
Figures S5 – S10
Tables S2 – S3

4) Henry’s law selectivity **8**
Figures S11 – S12
Tables S4 – S5

5) IAST selectivity **9**
Figures S13 – S18
Table S6

References **12**

1) Crystallographic data

Table S1. Summary of crystallographic experimental data.

Structure	**1-DMF_sq**	**1-EtOH_sq**	**1-THF**
Formula moiety	$Cu_2(C_8H_4O_4)_2\cdot(C_3H_7NO)_3(H_2O)_3$	$Cu_2(C_8H_4O_4)_2(C_2H_6O)_2\cdot(C_2H_6O)_{1.5}$	$Cu_2(C_8H_4O_4)_2(C_4H_8O)\cdot(C_4H_8O)_2$
Formula sum	$C_{25}H_{35}Cu_2N_3O_{14}$	$C_{23}H_{29}Cu_2O_{11.5}$	$C_{28}H_{32}Cu_2O_{11}$
M_r	728.65	616.54	671.61
Temperature (K)	120(2)	120(2)	120(2)
Crystal system	Monoclinic	Monoclinic	Monoclinic
Space group	$I2/m$	$I2/m$	$P2_1/n$
a (Å)<br>b (Å)<br>c (Å)	7.7935(5)<br>24.540(2)<br>16.1842(16)	6.8089(9)<br>25.808(3)<br>18.208(4)	8.0708(9)<br>25.437(3)<br>14.115(3)
β (°)	92.097(8)	89.805(16)	96.986(13)
V (Å^3)	3093.2(4)	3199.5(8)	2876.4(7)
Z	4	4	4
M (mm^{-1})	2.15	2.07	2.34
Crystal size (mm)	0.10 × 0.06 × 0.05	0.18 × 0.15 × 0.07	0.10 × 0.08 × 0.04
Radiation type	Cu $K\alpha$	Cu $K\alpha$	Cu $K\alpha$
Diffractometer, detector	SuperNova, Atlas	SuperNova, TitanS2	SuperNova, TitanS2
T_{min}, T_{max}	0.874, 0.963	0.973, 0.989	0.987, 0.995
Reflections collected	9248	5216	17278
Independent reflections	3171	1982	5694
Reflections [$I > 2\sigma(I)$]	2214	1371	2948
R_{int}	0.037	0.069	0.137
θ_{max} (°)	75.2	54.2	74.3
$\sin(\theta/\lambda)_{max}$ (Å^{-1})	0.627	0.526	0.624
$R[F^2 > 2\sigma(F^2)]$, $wR(F^2)$, S	0.091, 0.297, 1.16	0.171, 0.483, 1.85	0.101, 0.312, 1.01
No. of reflections	3171	1982	5694
No. of parameters	257	225	370
No. of restraints	718	639	90
$\Delta\rho_{max}$, $\Delta\rho_{min}$ (e Å^{-3})	1.11, −0.57	2.52, −0.71	1.27, −1.26
CCDC number	1558207	1558208	1558209

2) Analysis of bulk material

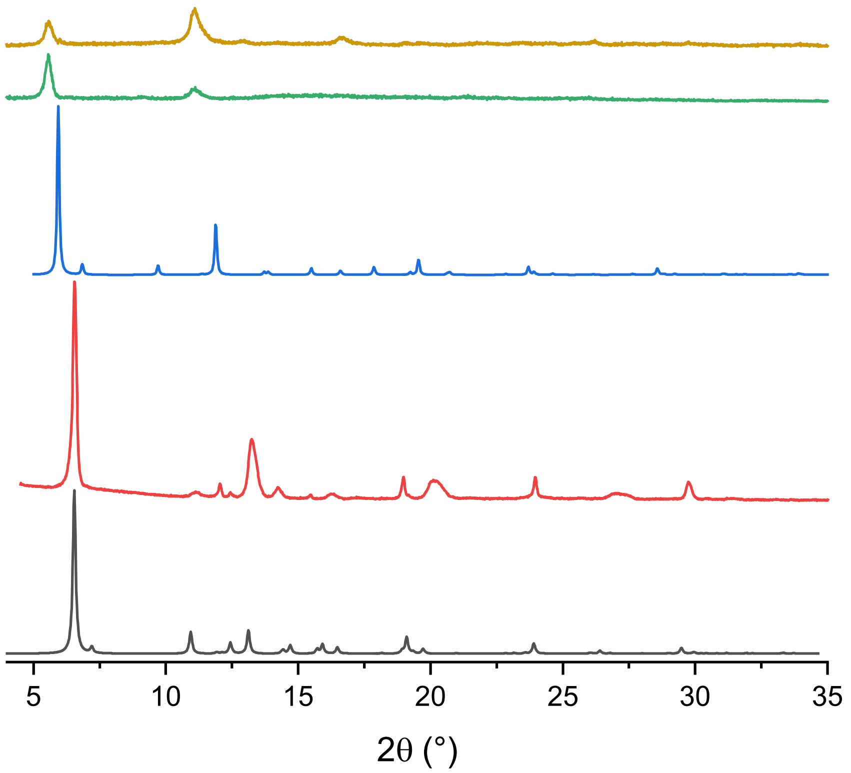

Figure S1. PXRD patterns of (bottom, black) simulated and (second from bottom, red) experimental as-synthesised **1-DMF**, (middle, blue) simulated **1-EtOH**, (second from top, green) activated **1-EtOH**, and (top, golden) **1** after gas sorption studies.

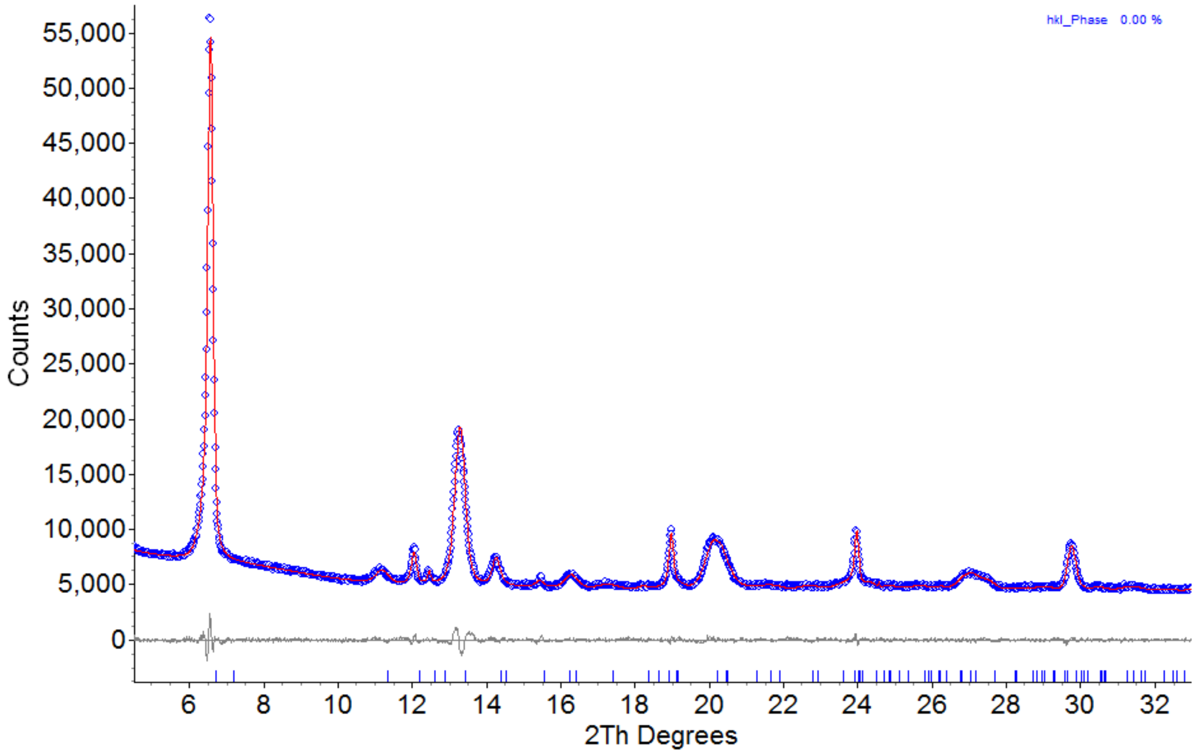

Figure S2. Fit from Pawley refinement[1] (Rwp = 2.10063901, Rexp = 1.2912527, GOF = 1.62682255): blue open circles = Y_{obs}, red line = Y_{calc} and grey line = difference.

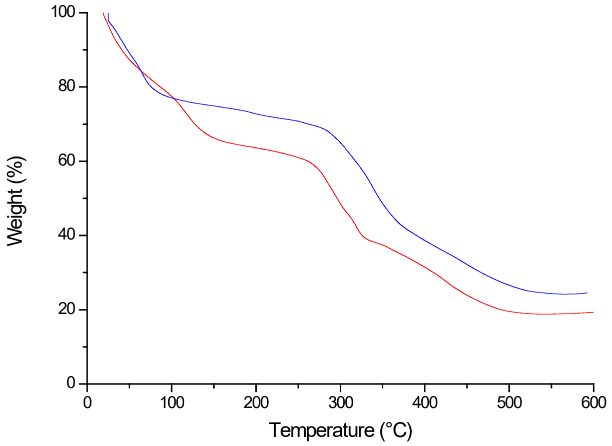

Figure S3. TGA of (red) as-synthesised **1-DMF** and (blue) ethanol exchanged **1-EtOH** under N_2 atmosphere.

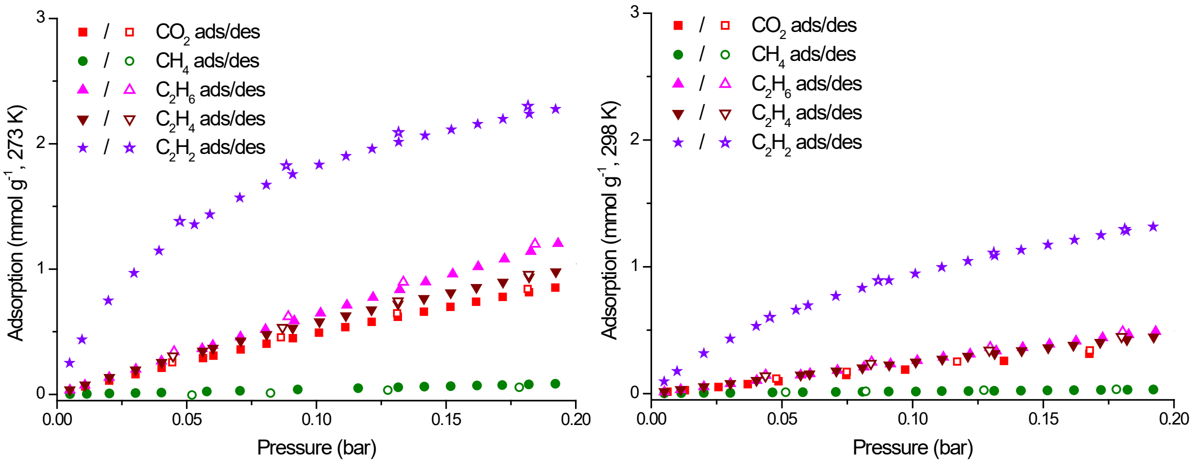

Figure S4. Adsorption and desorption isotherms of **1** for CO_2, CH_4 and the C_2H_n hydrocarbons at (left) 273 K and (right) 298 K between 0.0-0.2 bar.

3) Q_{st} calculations

To calculate the isosteric heat of adsorption (Q_{st}), first the adsorption isotherms from two different temperatures are compared using Equation 1,[2] where P is pressure (bar), n is amount adsorbed (mol g^{-1}), T is temperature (K) and a_i and b_j are virial coefficients related to temperature independent empirical parameters. The values m and o represent the number of coefficients used to fit the curves.

$$\ln(P) = \ln(n) + \left(\frac{1}{T}\right)\sum_{i=0}^{m} a_i n^i + \sum_{j=0}^{o} b_j n^j \tag{1}$$

The Q_{st} is calculated using Equation 2,[3] where R is the ideal gas constant, and can be plotted as a function of loading with the value at zero loading quoted.

$$Q_{st} = -R\sum_{i=0}^{m} a_i n^i \tag{2}$$

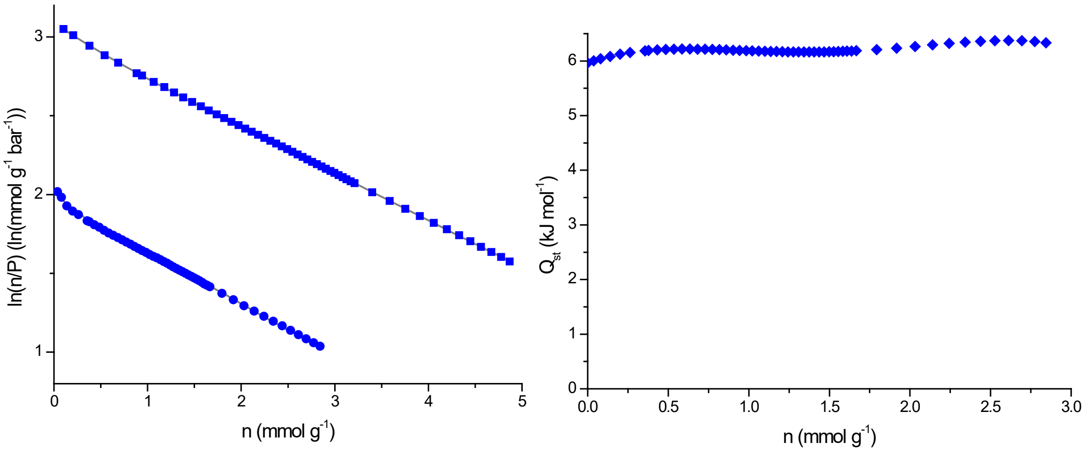

Figure S5. Plots of (left) ln(n/P) *vs.* n at (squares) 77 K and (circles) 87 K with virial fitting curves and (right) variation of Q_{st} for H_2 adsorption by **1**.

Table S2. Summary of the parameters obtained from the virial equation for H_2 adsorption by **1**.

	77 K	87 K
A_0	3.08753	2.01652
A_1	−0.39690	−0.58400
A_2	0.05599	0.31278
A_3	−0.01299	−0.14410
A_4	0.00106	0.02254
R^2	0.99998	0.99936

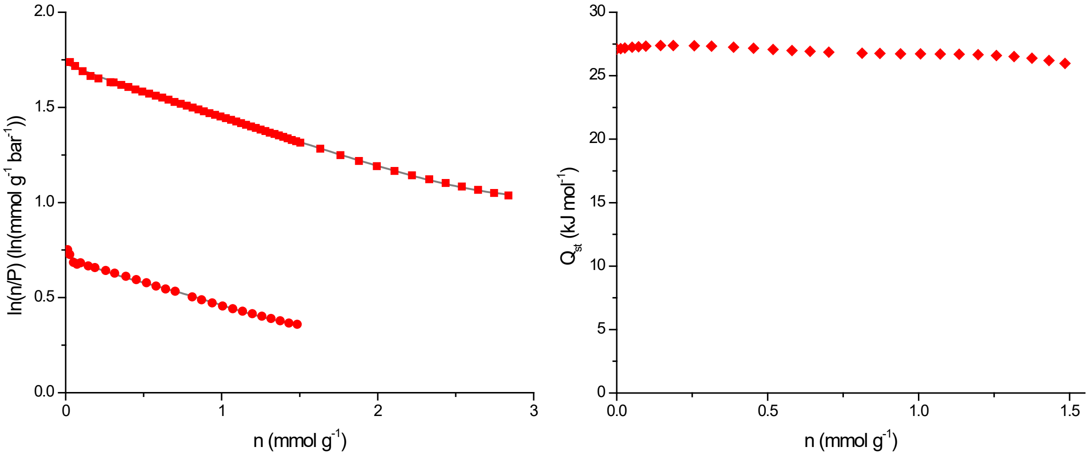

Figure S6. Plots of (left) ln(n/P) *vs.* *n* at (squares) 273 K and (circles) 298 K with virial fitting curves and (right) variation of Q_{st} for CO_2 adsorption by **1**.

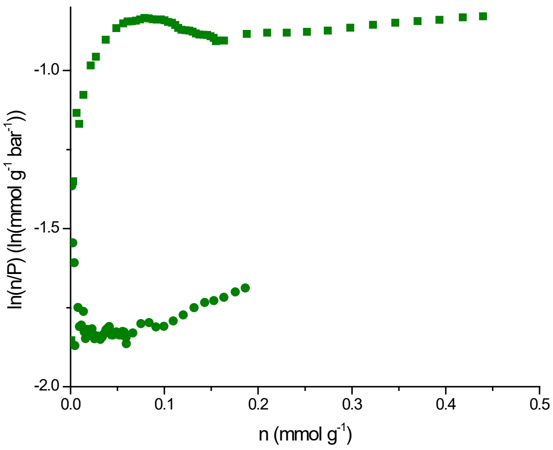

Figure S7. Plots of ln(n/P) *vs.* *n* at (squares) 273 K and (circles) 298 K for CH_4 adsorption by **1** (virial fitting not performed for this data).

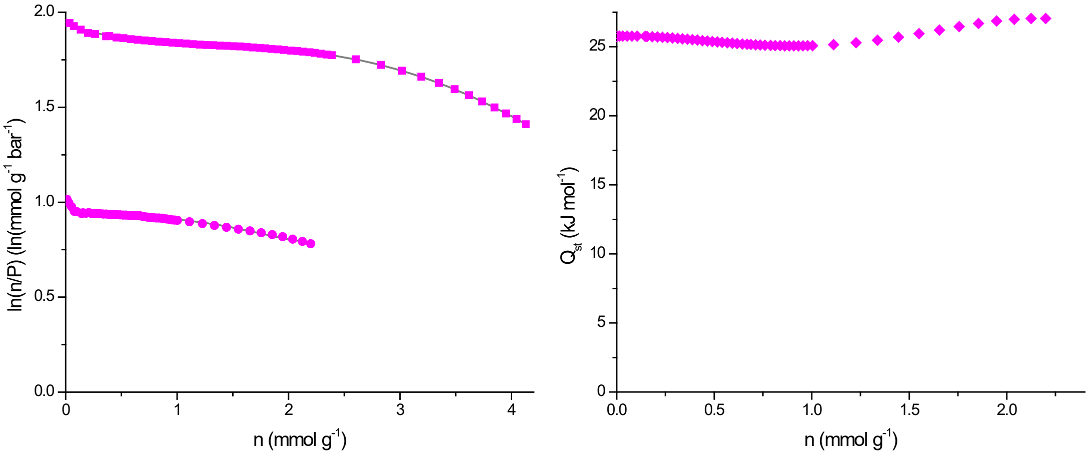

Figure S8. Plots of (left) ln(n/P) *vs.* *n* at (squares) 273 K and (circles) 298 K with virial fitting curves and (right) variation of Q_{st} for C_2H_6 adsorption by **1**.

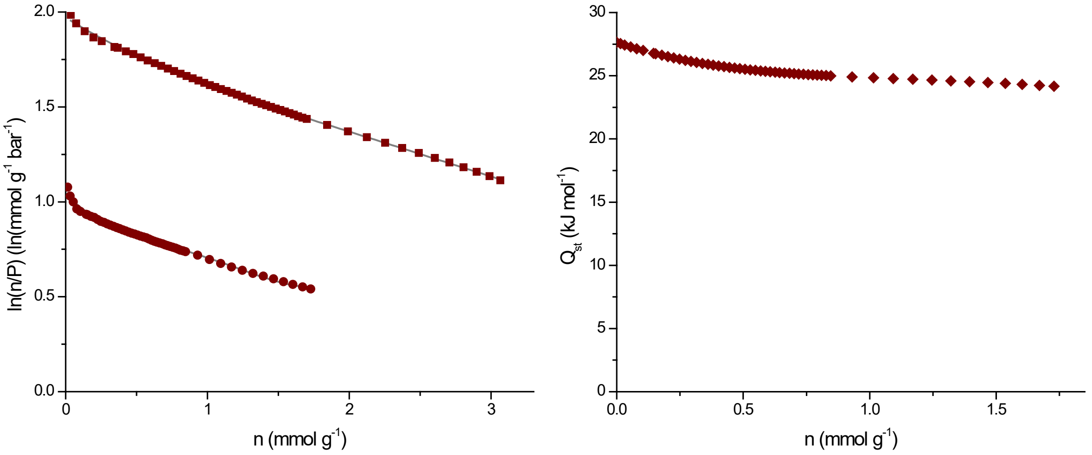

Figure S9. Plots of (left) ln(n/P) *vs.* *n* at (squares) 273 K and (circles) 298 K with virial fitting curves and (right) variation of Q_{st} for C_2H_4 adsorption by **1**.

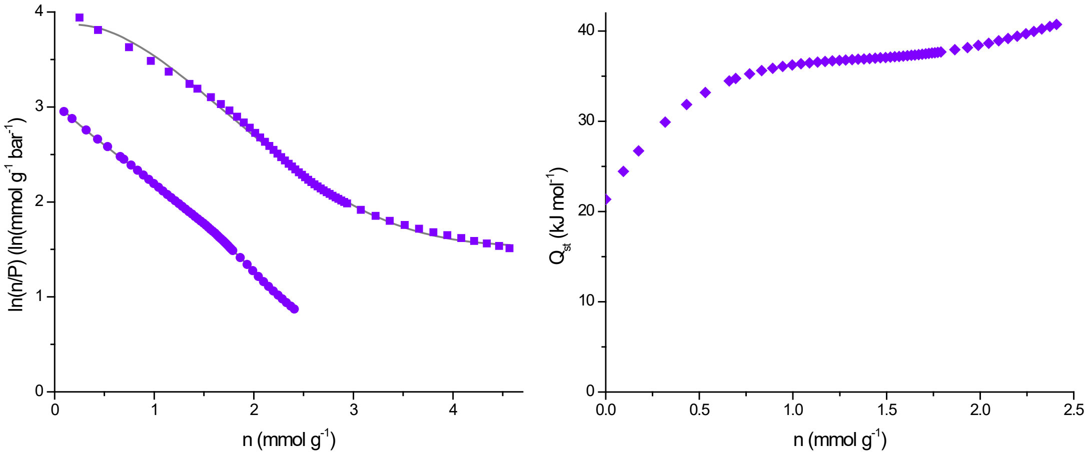

Figure S10. Plots of (left) ln(n/P) *vs.* *n* at (squares) 273 K and (circles) 298 K with virial fitting curves and (right) variation of Q_{st} for C_2H_2 adsorption by **1**.

Table S3. Summary of the parameters obtained from the virial equation for CO_2 and C_2H_n hydrocarbon adsorption by **1**.

	CO_2		C_2H_6		C_2H_4		C_2H_2	
	273 K	298 K	273 K	298 K	273 K	298 K	273 K	298 K
A_0	1.72983	0.72823	1.93574	0.98350	1.97054	1.04223	3.84304	3.05330
A_1	−0.33563	−0.45876	−0.19765	−0.21679	−0.46812	−0.76711	0.26736	−1.04538
A_2	0.09563	0.52708	0.13596	0.30379	0.15489	0.95662	−0.75709	0.37618
A_3	−0.05263	−0.49604	−0.04155	−0.20410	−0.04531	−0.71099	0.19936	−0.22521
A_4	0.01075	0.15986	0.00309	0.04207	0.00490	0.18455	−0.01544	0.03816
R^2	0.99940	0.99324	0.99928	0.96624	0.99899	0.99351	0.99691	0.99976

4) Henry's law selectivity

Equation 3,[4] of virial form, is used to fit the adsorption isotherm data, where n is the amount adsorbed (mol g^{-1}), P is pressure (Pa) and A_0, A_1, *etc.* are virial coefficients.

$$\ln\left(\frac{n}{P}\right) = A_0 + A_1 n + A_2 n^2 + A_3 n^3 + ... \quad (3)$$

The coefficient A_0 is used to calculate k_H by Equation 4.[5]

$$k_H = e^{A_0} \quad (4)$$

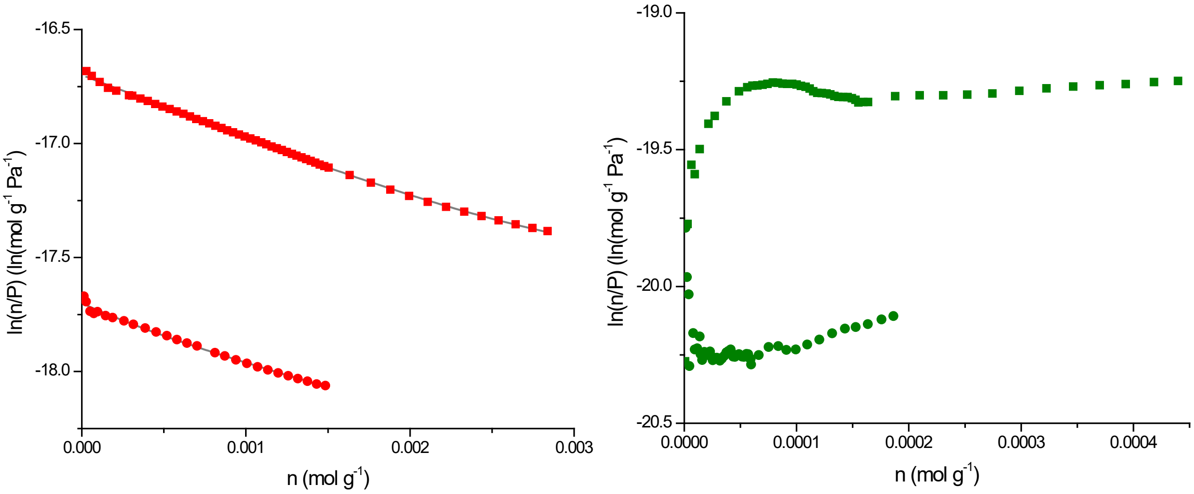

Figure S11. Plots of ln(n/P) *vs.* n at (squares) 273 K and (circles) 298 K for (left) CO_2 and (right) CH_4 adsorption by **1**. CH_4 data not fitted to Henry's law.

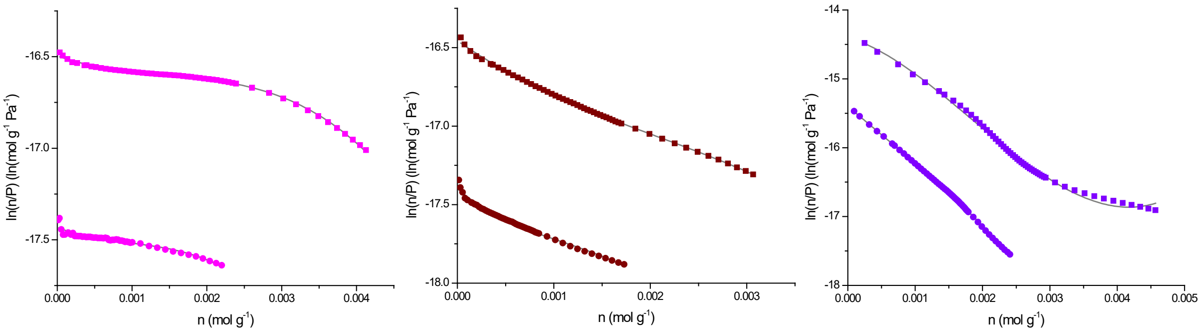

Figure S12. Plots of ln(n/P) *vs.* n at (squares) 273 K and (circles) 298 K for (left) C_2H_6, (centre) C_2H_4 and (right) C_2H_2 adsorption by **1** fitted to Henry's law.

Table S4. Summary of the parameters for fittings to calculate selectivity using Henry's law for CO_2 and C_2H_n hydrocarbon adsorption by **1**.

		CO_2	C_2H_6	C_2H_4	C_2H_2
273 K	A_0	−16.70014	−16.49624	−16.45622	−14.39469
	R^2	0.99905	0.99791	0.99891	0.99526
298 K	A_0	−17.70019	−17.43331	−17.39895	−15.39712
	R^2	0.99222	0.93124	0.98918	0.99966

Table S5. Selectivities between uptake of C_2H_n hydrocarbons by **1** determined *via* comparison of k_H values.

	C_2H_4/C_2H_6	C_2H_2/C_2H_6	C_2H_2/C_2H_4	C_2H_2/CO_2
273 K	1.03 : 1	8.18 : 1	7.86 : 1	10.00 : 1
298 K	1.04 : 1	7.66 : 1	7.40 : 1	9.81 : 1

5) IAST selectivity

The experimental single-component adsorption data has been fitted to the single site or dual site Langmuir-Freundlich model (Equation 5),[6] where n is the total amount adsorbed (mmol g^{-1}), P is pressure (bar), q_{sat} is the saturation capacity of each site (mmol g^{-1}), b is the Langmuir parameter for each site (bar^{-1}), v is the Freundlich parameter for each site and 1 and 2 denote the sites.

$$n = \frac{q_{sat_1} b_1 P^{v_1}}{1 + b_1 P^{v_1}} + \frac{q_{sat_2} b_2 P^{v_2}}{1 + b_2 P^{v_2}} \tag{5}$$

For each gas, both isotherms (273 K and 298 K) were fitted simultaneously with shared q_{sat} and v parameters and a single site model was adopted where possible. In all instances the good agreement of the modelling with the data is highlighted by R^2 values exceeding 0.999. The selectivity is defined in Equation 6, where S is the selectivity factor, x is the amount adsorbed determined by IAST, y is the mole fraction of the adsorbate in the gas phase at equilibrium and i and j denote different adsorbates.

$$S = \frac{x_i / y_i}{x_j / y_j} \tag{6}$$

Isotherms were obtained by applying the IAST method for binary systems of 50:50 concentration up to a total pressure of 1 bar at 273 K and 298 K.

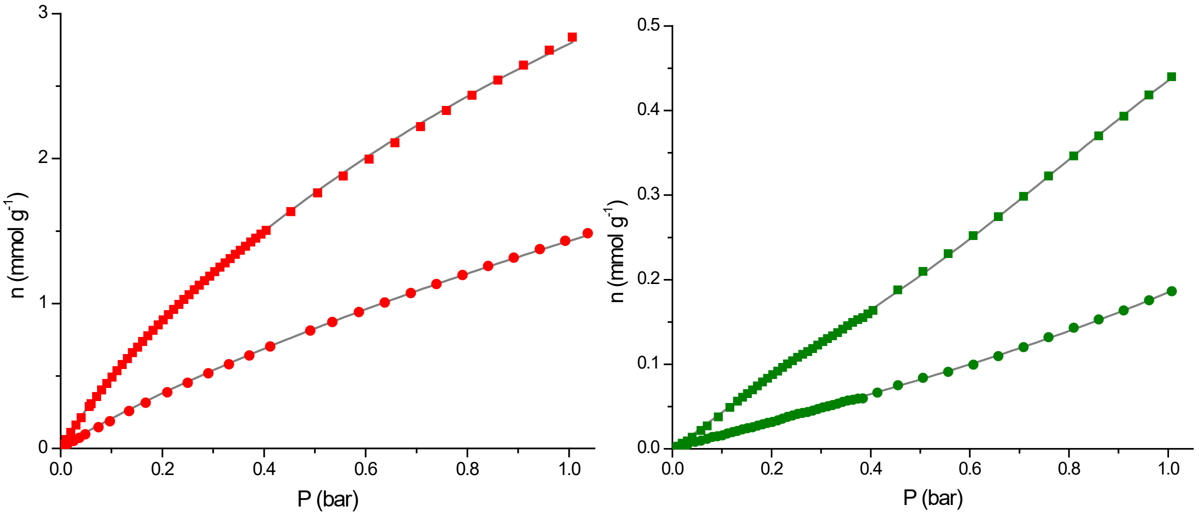

Figure S13. Adsorption isotherms of (left) CO_2 fitted to the single site Langmuir-Freundlich model and (right)

CH_4 fitted to the dual site Langmuir-Freundlich model at (squares) 273 K and (circles) 298 K.

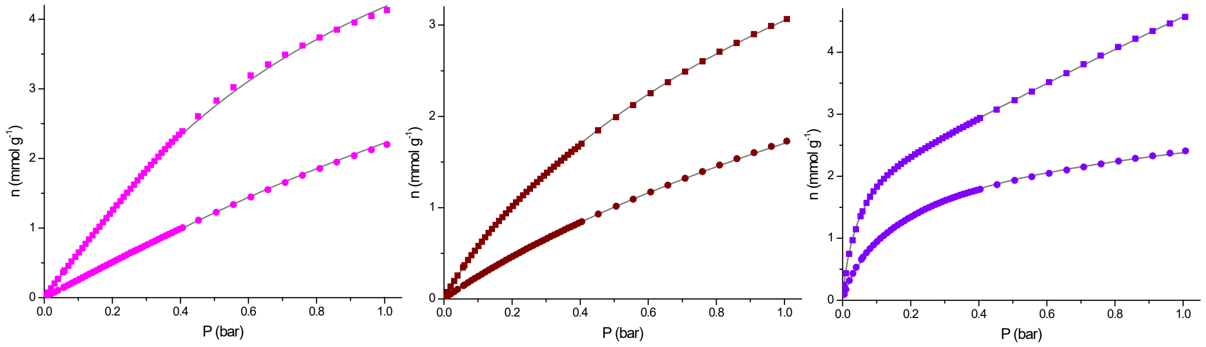

Figure S14. Adsorption isotherms of (left) C_2H_6 and (centre) C_2H_4 fitted to the single site Langmuir-Freundlich model and (right) C_2H_2 fitted to the dual site Langmuir-Freundlich model at (squares) 273 K and (circles) 298 K.

Table S6. Summary of the parameters for the simultaneous fitting of the isotherms for each adsorbate with shared q_{sat} and v parameters.

	CO_2		CH_4		C_2H_6		C_2H_4		C_2H_2	
	273 K	298 K	273 K	298 K	273 K	298 K	273 K	298 K	273 K	298 K
q_{sat_1}	8.18002	8.18002	0.54438	0.54438	7.55230	7.55230	7.60055	7.60055	2.90522	2.90522
b_1	0.51796	0.21208	1.07261	0.35756	1.23848	0.41701	0.67073	0.28923	11.14758	3.43928
v_1	0.91371	0.91371	1.09802	1.09802	1.11734	1.11734	0.92392	0.92392	0.86767	0.86767
q_{sat_2}	-	-	0.32996	0.32996	-	-	-	-	7.11494	7.11494
b_2	-	-	0.87125	0.14394	-	-	-	-	0.36509	0.01843
v_2	-	-	3.53046	3.53046	-	-	-	-	1.69712	1.69712
R^2	0.99977		0.99982		0.99937		0.99992		0.99976	

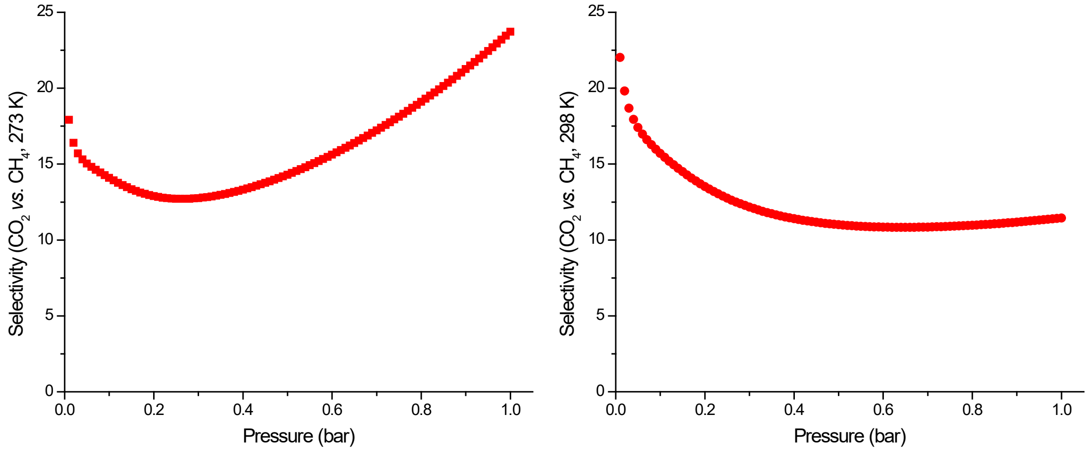

Figure S15. Selectivity of adsorption of CO_2/CH_4 by **1** at (left) 273 K and (right) 298 K, calculated for a 50:50 mixture using the IAST method.

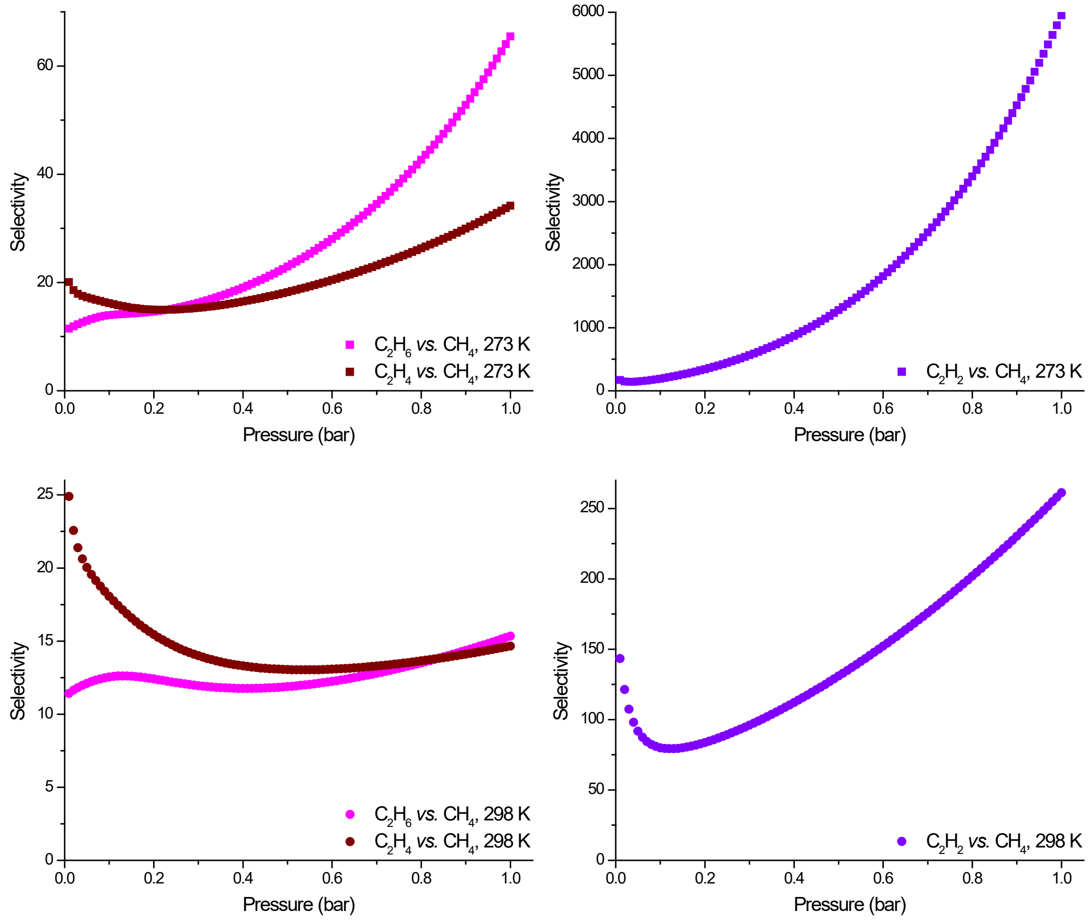

Figure S16. Selectivity for adsorption of C_2H_n/CH_4 by **1** at (top) 273 K and (bottom) 298 K, calculated for 50:50 mixtures using the IAST method.

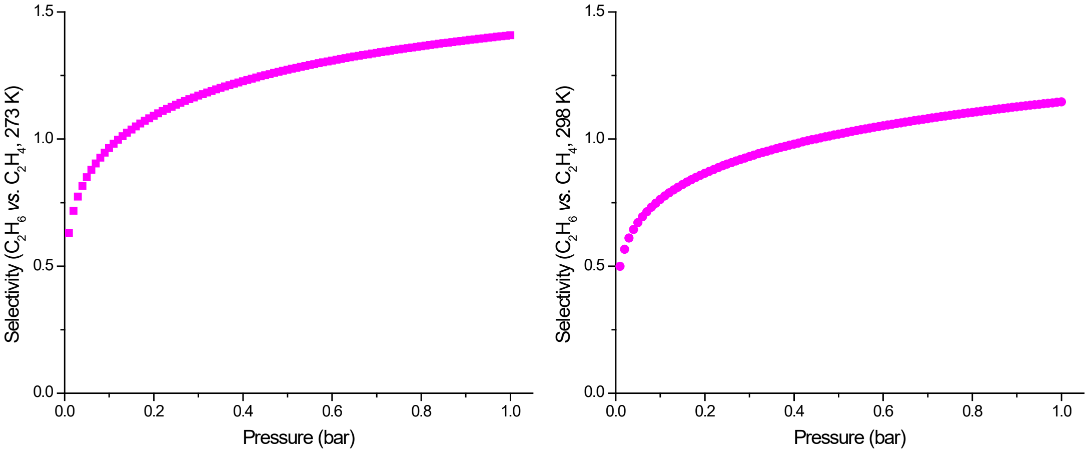

Figure S17. Selectivity of adsorption of C_2H_6/C_2H_4 by **1** at (left) 273 K and (right) 298 K, calculated for 50:50 mixtures using the IAST method.

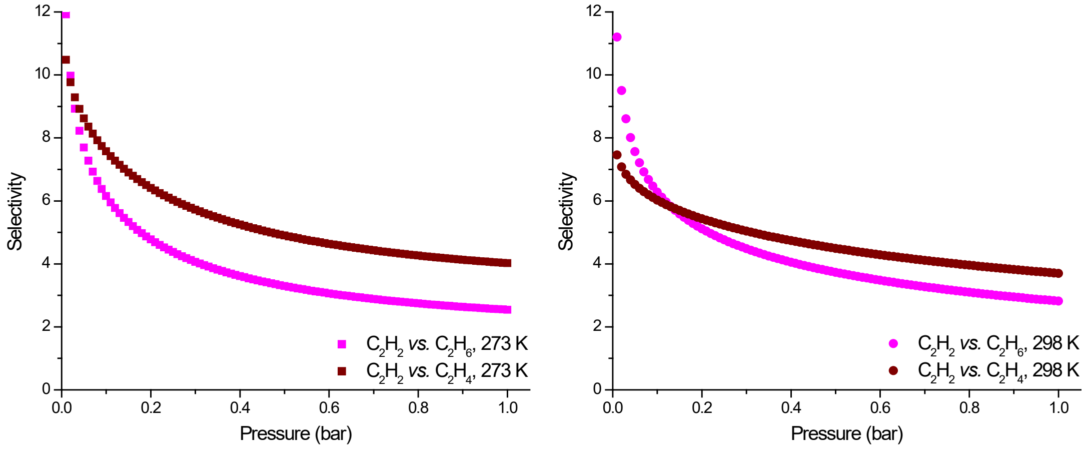

Figure S18. Selectivity of adsorption of C_2H_2/C_2H_n by **1** at (left) 273 K and (right) 298 K, calculated for 50:50 mixtures using the IAST method.

References

[1] G. S. Pawley, *J. Appl. Crystallogr.*, **1981**, *14*, 357.

[2] a) L. Czepirski, J. Jagiełło, *Chem. Eng. Sci.*, **1989**, *44*, 7971; b) J. Jagiełło, T. J. Bandosz, J. A. Schwarz, *Langmuir*, **1996**, *12*, 2837; c) I. P. O'koye, M. Benham, K. M. Thomas, *Langmuir*, **1997**, *13*, 4054; d) C. R. Reid, I. P. O'koye, K. M. Thomas, *Langmuir*, **1998**, *14*, 2415; e) C. R. Reid, K. M. Thomas, *Langmuir*, **1999**, *15*, 3206.

[3] a) A. Ansón, J. Jagiello, J. B. Parra, M. L. Sanjuán, A. M. Benito, W. K. Maser, M. T. Martínez, *J. Phys. Chem. B*, **2004**, *108*, 15820; b) B. Chen, X. Zhao, A. Putkham, K. Hong, E. B. Lobkovsky, E. J. Hurtado, A. J. Fletcher, K. M. Thomas, *J. Am. Chem. Soc.*, **2008**, *130*, 6411.

[4] a) C. R. Reid, K. M. Thomas, *J. Phys. Chem. B*, **2001**, *105*, 10619; b) X. B. Zhao, B. Xiao, A. J. Fletcher, K. M. Thomas, *J. Phys. Chem. B*, **2005**, *109*, 8880.

[5] J. H. Cole, D. H. Everett, C. T. Marshall, A. R. Paniego, J. C. Powl, F. Rodriguez-Reinoso, *J. Chem. Soc. Faraday Trans. 1 Phys. Chem. Condens. Phases*, **1974**, *70*, 2154.

[6] R. T. Yang, *Gas Separation by Adsorption Processes*, Imperial College Press, London, **1997**.
